# The burden of *Neisseria gonorrhoeae* infection, risky sexual behavior, and associated risk factors among sexually transmitted infections in a resource-limited setting area of Addis Ababa City, Ethiopia

**DOI:** 10.3389/frph.2025.1601088

**Published:** 2025-08-29

**Authors:** Tesfaye Andualem, Gurja Belay, Adey F. Desta, Helen Nigussie, Wondemagegn Mulu, Asnake Desalegn, Gizachew Taddesse, Yonas Mekonen, Degefu Beyene

**Affiliations:** ^1^ Department of Medical Laboratory Science, College of Health Science, Debre Tabor University, Gondar, Ethiopia; ^2^ Department of Microbial Sciences and Genetics, College of Natural and Computational Sciences, Addis Ababa University, Addis Ababa, Ethiopia; ^3^ Department of Medical Laboratory Sciences, College of Medicine and Health Sciences, Bahir Dar University, Bahir Dar, Ethiopia; ^4^ Department of Microbiology, Immunology, and Parasitology, School of Medicine, St. Paul’s Hospital Millennium Medical College, Addis Ababa, Ethiopia; ^5^ Department of Clinical Microbiology, and Mycology, Ethiopian Public Health Institute, Addis Ababa, Ethiopia

**Keywords:** gonorrhea, *N. gonorrhoeae*, risk factors, risky sexual behavior, sexually transmitted infection

## Abstract

**Introduction:**

*N. gonorrhoeae* is the cause of gonorrhea, which is one of the most common public health problems among sexually transmitted infections. The highest incidence of disease occurs in less developed countries, and gonococcal infections are common among adolescents and young adults. Risky sexual behavior (RSB) is also the main concern. It has many consequences on the health system, which is the most risk factor for the transmission of sexually transmitted diseases, particularly gonorrheal diseases. Little is known about the magnitude of gonococcal infection and risky sexual behavior (RSB) in the reproductive age groups.

**Objectives:**

To assess the burden of *Neisseria gonorrhoeae*, Risky Sexual Behavior, and Associated Risk Factors among Sexually Transmitted Infections in a Resource-Limited Area of Addis Ababa City, Ethiopia.

**Methods:**

A health institution-based cross-sectional study was conducted from April 2023 to December 2024 in Addis Ababa City. A convenient sampling method was used to collect endocervical and urethral sample swabs from 571 study subjects. Samples were cultured onto Thayer Martin Luther agar, and gram staining and biochemical tests were used to confirm the presence of gonococci. A pre-tested and well-structured questionnaire was used to assess risk factors, and data were analyzed using SPSS version 22. Descriptive and logistic analyses were computed. *P*-values ≤0.05 were considered statistically significant.

**Results:**

Of the total study subjects, 62.2% were females, and 61.6% were urban residents. Moreover, 183 (32.0%) were in the age of >35 years, followed by 170 (29.8%) in the 30–34 years old. The prevalence of *N. gonorrhoeae* among STI patients was 17.33%, and risky sexual behavior was 56.9%. The odds of *N. gonorrhoeae* infection were 1.55 times higher among chat users than the non-chat users [AOR = 1.55, 95% CI: (1.32–1.95)]. Similarly, the odds of risky sexual behavior were 10.95 [AOR = 10.95, 95% CI (5.75–20.84)] times higher among STIs who had a new sexual partner than their counterparts.

**Conclusion:**

The prevalence of *N. gonorrhoeae* and risky sexual behavior among STI patients were high. Gender, watching pornographic films, alcohol consumption, and not participating in religious education have been found to increase the risk of experiencing both *N. gonorrheae* infections and risky sexual behavior.

## Introduction

Sexually transmitted infections (STIs) have been the major causes of morbidity and mortality for many years and are predominantly transmitted via sexual contact. Despite the medical system becoming advanced, STIs continue to pose a threat to health and are a major public health problem with an annual estimate of 374 million people infected with them (1). To address these public health issues, the WHO has adopted a strategy to control STIs that aligns with the Agenda for the Sustainable Development Goals (SDGs) by 2030. To minimize the incidence and prevalence of STIs, targeting the population at greater risk, effective clinical interventions, promoting the use of condoms, and having reliable data are useful means (1, 2).

*Neisseria gonorrhoeae* (NG*)* is one of the most common STIs, which causes gonorrhea. The bacterium is a gram-negative, oxidase-positive, non-spore-forming, non-capsulated, kidney-shaped diplococcus. It affects adolescents and infects the newborn infant during delivery (1, 3). According to the World Health Organization (WHO) 2016 report, gonorrhea cases were 30.6 million worldwide, with a prevalence of 0.9% females and 0.7% in males. The highest prevalence of gonorrhea among females and males in the Africa WHO region was 1.9% and 1.6%, respectively (4), whereas a study in sub-Saharan Africa indicated that the pooled prevalence was 2.4% in females and 1.7% in males (5). The summary report of a WHO European region from 1949 to 2021 revealed that the pooled prevalence of gonococcus (GC) among females and males was 3.2% and 12.1%, respectively (6).The disease is one of the main public health problems in Ethiopia. Some of the recent studies in Ethiopia indicated that the pooled prevalence of gonorrhea among STI-suspected patients ranged from 0.4% to 69% (7, 8), Mekelle (10.4%) (9), Gondar (7.6%) (10), Addis Ababa (50%) (11), Hawassa (4.3%) (12), and Jimaa (9.8%) (13). Even though females are more vulnerable to infections during sexual contact than males, the gonorrhea rates in males are mostly higher than in females. That is due to the lower rates of asymptomatic disease in females than in males. It infects the mucosal surfaces of the cervix in women and the urethra in men (4, 14).

The more likely high-risk groups for the infection of gonorrhea are commercial sex workers, those in more densely populated areas, low socioeconomic status, young individuals aged less than 25, and migrants. The main risk factors for acquiring STIs are unsafe sex practices, multiple sexual partners, substance use, early sexual intercourse, and risky sexual behavior (14–16).

Risky sexual behaviors (RSB) are behaviors that include engaging in sexual activity from an early age, inconsistent use of condoms during sexual intercourse, unprotected sexual intercourse, having sex with commercial sex workers, and the tendency to have multiple sexual partners that enhance the transmission of STIs including NG (10, 17). A recent systematic review of RSB in sub-Saharan Africa showed that the prevalence was 36.16% (18), whereas the pooled prevalence in Ethiopia was 42.8% (19). The report of the Ethiopian Demographic and Health Survey (EDHS) indicated that the average percentage of first sexual intercourse in the age group of 18–24-year-olds in 2019 was 40%, and 12% for females and males, respectively (20). The Ethiopian government's Ministry of Health has developed, implemented, and introduced various strategies to prevent and control sexual and reproductive health. For effective prevention and care of risky sexual behavior and STIs, there have been monitoring and evaluation methods (21).

As indicated, the prevalence of *N. gonorrhoeae* and risky sexual behavior in Ethiopia showed that there was a significant variation in their distributions across the regions. That was due to the availability and/or unavailability of the laboratory settings, the recommended type of culture media, the growth supplements, and the inhibitors for the cultivation of *N. gonorrhoeae* in each diagnostic laboratory. Addis Ababa is one of the regions where the prevalence of STIs is very high (8). It is commonly assumed that urban residents are more educated, have awareness and information, and based on that, they can practice. As a result, they are considered the low-risk population compared to their counterparts. However, practical observation and many articles indicated that the opposite happens (22, 23).

However, the risk factors are common in the city; there is no adequate information on the magnitude of risky sexual behaviors and *N. gonorrhoeae.* Therefore, this study was done to determine the prevalence of *N. gonorrhoeae*, risky sexual behavior, and associated risk factors among sexually transmitted infections in a resource-limited area of Addis Ababa City, Ethiopia.

## Materials and methods

### Study design and setting

A health institution-based cross-sectional study was conducted among sexually transmitted infection-suspected patients from April 2023 to December 2024 at the Addis Ababa City administration, the capital of Ethiopia. The city has over six million residents, twenty one governmental hospitals, and one hundred three health centers.

### Sample size determination

A single proportional formula was used to calculate the sample size with a 95% confidence interval, a 4% margin of error, and an 11% contingency. A 69% (8) prevalence from the previous study in Ethiopia was used, and the final estimated sample was 571.

Using a 95% confidence interval with a 4% margin of error sample size was calculated as follows.

$N=\frac{Z2\;P(1-p)}{W^{2}}$

Where:

*n* = No of samples that will be included

$\frac{Z2}{2}$ = confidence level

P = prevalence from the previous study.

W = margin of error

$$n=\frac{{\left(1.96\right)}^{2}\times 0.69\times 0.31}{{\left(0.04\right)}^{2}}=\frac{0.8214}{0.0016}=514+11\text{\%}=571$$

### Study variables

The prevalence of *N. gonorrhoeae* and risky sexual behavior were the dependent variables.

The independent variables were the socio-demographic variables (sex, age, marital status, occupation, educational status, participation in religious education, use of a condom, living with a spouse), the substance-related factors (alcohol consumption, khat chewing, and cigarette smoking), and risky sexual behavior factors (watching pornographic movies, attending nightclubs, having a new sexual partner, having a sexual partner two or more, previous STI, previous STI drug use, and commercial sex workers).

### Sampling method and data collection

The study sites were Addis eray health center, Kuas meda health center, Millennium health center, Afenchobere health center, and Yeka health center. They were selected based on the previous STI - suspected patient flow in the health institutions, mass population living areas, and expected low living standards of the community near the health center. A convenient sampling technique was used to include the study subjects.

Clinicians and the primary investigator participated in the data collection, and sample processing was done via the principal investigator. The study subjects were those of all the reproductive age groups with any one of the signs and symptoms of gonorrhea who attended the health institutions during the study period. Individuals who had no signs and symptoms of STIs, were on recent antibiotic treatment, and were outside the reproductive age group were excluded from the study group. All eligible participants who attended the health center OPD were informed about the research objective and asked for their permission. After obtaining the participants' consent, a face-to-face interview using a structured questionnaire was conducted to collect the socio-demographic data and the risk factor variables related to GC and RSB.

### Specimen collection

Two sterile swabs were collected from the endocervical canal of females using a speculum with warm water, and urethral samples were collected from males. One swab was for gram staining, and the other was for culturing. During the collection of the samples from the females, swabs were inserted into the cervix up to 2–3 cm, rotated for 5–10 s, and urethral samples were collected directly from the discharges.

### Transport of the samples

After collecting the sample from the study subject, it was immediately delivered to the Amines transport media, which were suitable for 6–12 h of collection. The semisolid Amines transport media with charcoal can neutralize the toxic byproducts and other inhibitory substances and also contain sodium chloride to preserve these viable NG organisms. The samples were transported to the nearby reference laboratories of the Ethiopian Public Health Institute and the Institute of Biotechnology, Addis Ababa University. The transport media were labeled with the sample number, date, name of the health center, and time of collection. Ideally, the recommendation is that the samples of the NG are to be inoculated directly into their selective culture media to preserve the NG organisms. However, in this case, the sample collection sites were in different health centers, which had to be transported to the reference laboratories (14, 24).

### Isolation and identification of *N. gonorrhoeae*

#### Microscopy

In the reference laboratories, one swab was used for gram staining to look for a bean-shaped gram-negative diplococcus under a microscope, which was important for the presumptive diagnosis of NG.

#### Culture

Gonococci are fastidious organisms; they need an enriched culture medium to support their growth and development, and selectives to suppress the growth of other organisms. The swabs taken from individual patients were inoculated onto selective Modified Thayer-Martin medium (MTM) with the supplements and incubated at 37°C in a moist atmosphere enriched with 5%–10% CO2 for 24–48 h. The MTM media were supplemented with IsovitaleX to support the growth of NG and VCNT (vancomycin, colistin, nystatin, and trimethoprim) inhibitors for other organisms. Culture plates were examined within 18–24 h after incubation and again after 48 h of incubation. The positive cultures produced small, raised, shiny, grey colonies that were sub-cultured on enriched media of chocolate agar, and all positive cultures were identified by their characteristic appearance on the media. Suspected NG in culture media were identified using gram stain, oxidase test, superoxol test, and carbohydrate utilization tests.

Gram staining was performed on the suspected colonies from the culture media to confirm a kidney-shaped gram-negative diplococcus. In the oxidase test, a suspected colony was made into a smear on the filter paper, and a positive result showed purple color development. In the superoxol test (30% w/v hydrogen peroxide), the suspected colonies from the culture media were mixed with 30% w/v hydrogen peroxide, and positive results showed strong bubble formation. For the carbohydrate utilization test, the suspension of the Ng isolates from the culture media was added to each of the glucose, sucrose, and maltose tube media. The color change observed in the glucose media from red to yellow, but not in the sucrose and maltose media, was considered as Ng isolates. The isolated NG were stored at −80°C in tryptic soy broth (TSB) with glycerol (14, 24, 25).

#### Data quality

The reliability of the study findings was ensured by implementing quality control (QC) measures throughout the entire laboratory work process. All materials, equipment, and procedures were adequately enrolled, and culture media were tested for sterility and performance. Pre-analytical, analytical, and post-analytical stages of quality assurance that were incorporated in standard operating procedures (SOPs) of the Microbiology laboratory at the Institute of Microbiology laboratory were strictly followed. The standard reference strains of *N. gonorrhoeae* ATCC 49226 (26) were used as controls for culture and biochemical tests (26).

#### Data analysis

Data were entered and analyzed using SPSS statistical software version 22. Frequencies and cross-tabulations were used to summarize descriptive statistics. Tables were used for data presentation. The odd ratio and adjusted odds ratio, both bivariate and multiple logistic regression, were employed to assess the association between outcome and explanatory variables. *P*-values <0.05 were considered statistically significant.

### Operational definition

#### Commercial sex worker

Study participants who had sex for the exchange of money on commercial sites, hotels, streets, and residences (27).

#### Early sexual intercourse

Study participants who had had sexual intercourse before the age of 18 years old (28).

#### Illiterate

Individuals who didn't attend formal education

#### Literate

Individuals who attended formal education.

#### Multiple sexual partners

Study participants who had sexual intercourse with two or more sexual partners (19).

#### Risky sexual behavior (RSB)

Those who are reported having sexual intercourse with multiple sexual partners in the past year, sex with commercial sex workers, early initiation of sex, and unprotected sex (19).

#### Sexual experience

Having sexual intercourse practice in their life.

#### Substance use

Individuals who have used these substances in the last year (alcohol, khat, and cigarette use) in any amount (29).

### Ethical considerations

Ethical clearance was obtained from the ethical review committee of the College of Natural and Computational Sciences, Addis Ababa University, with reference no CNCSDO/622/15/2023. Written informed consent was obtained from the study participants. The study was conducted following the Declaration of Helsinki.

## Result

### Socio-demographic characteristics of the study participants

The study includes 571 suspected STI patients in Addis Ababa City from April 2023 to December 2024, ranging from 15 to 67 years. Most of the study participants were in the age group of >35 years (32%), followed by 30–34 years (29.8%), with a mean age of 32.76 years (SD 8.24). Moreover, many participants were from an urban area 352 (61.6%). Three hundred twenty (56.0%) were unmarried, 459 (80.4%) were literate, and 329 (57.6%) were unemployed (Table 1).

**Table 1:** Socio-demographic characteristics of the study participants among STI-suspected patients (*n* = 571) in Addis Ababa, Ethiopia, 2024.

Socio-demographic	*N. gonorrhoeae* infection		COR (95 CI	*P*-value	AOR (95 CI)	*P*-value
Characteristics *n* (%)	Positive (*n* = 99) *n* (%)	Negative *n* = 472 *n* (%)				
Gender						
Male (216) (37.8)	54 (9.5)	162 (28.4)	0.44 (0.28–0.68)	<.001	0.412 (0.26–0.65)	<.001
Female (355) (62.2)	45 (7.9)	310 (54.3)	1		1	
Age						
18–19 (8) (1.4)	1 (0.2)	7 (1.2)	1.32 (0.16–11.12)	0.8		
20–24 (71) (12.4)	15 (2.6)	56 (9.8)	0.70 (.35–1.41)	0.32		
25–29 (139) (24.3)	22 (3.9)	117 (20.5)	1.00 (.55–1.83)	0.99		
30–34 (170) (29.8)	32 (5.6)	138 (24.2)	0.81 (.47–1.41)	0.46		
>35 (183) (32.0)	29 (5.1)	154 (27)	1			
Resident						
Rural (219) (38.4)	53 (9.3)	166 (29.1)	2.12 (1.37–3.29)	0.001	2.34 (1.48–3.69)	0001
Urban (352) (61.6)	46 (8.1)	306 (53.6)				
Marital status						
Unmarried (320) (56.0)	64 (11.2)	256 (44.8)	0.65 (0.41–1.02)	0.059	0.78 (0.48–1.24)	0.29
Married (251) (44.0)	35 (6.1)	216 (37.8)	1			
Educational status						
Illiterate (112) (19.6)	16 (2.8)	96 (16.8)	1	0.342		
Literate (459) (80.4)	83 (14.5)	376 (65.8)	1.32 (0.74–2.37)			
Occupation						
Unemployed (329) (57.6)	68 (11.9)	261 (45.7)	1.77 (1.12–2.81)	0.015	0.68 (0.422–1.09)	0.113
Employed (242) (42.4)	31 (5.4)	211 (37.0)	1		1	

### Prevalence of *N. gonorrhoeae* infection

**Figure 1:**
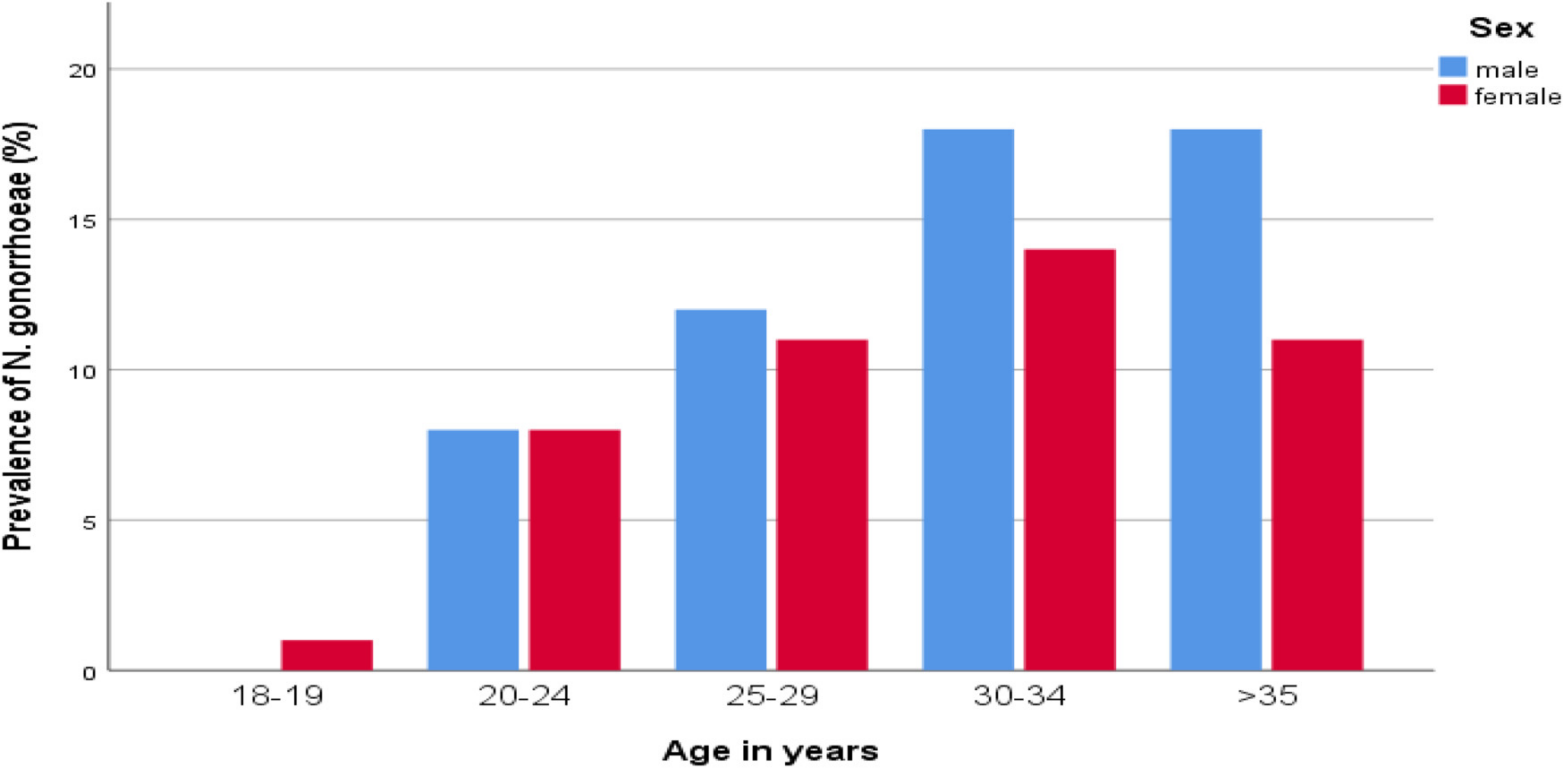
Distribution of *N. gonorrhoeae* by age groups and sex of STI suspected patients in Addis Ababa, Ethiopia.

Of the 571 study participants, 99 (17.33%) had a confirmed gonococcal (GC) infection. The highest prevalence of GC infection was observed among those aged 30–34 years (5.6%), followed by those older than 35 years (5.1%), compared to a low occurrence in the 15–19 age group (0.2%) (Figure 1). The rate of GC infections was lower among married individuals (6.1%) than among unmarried individuals (11.2%), and among literate participants (14.5%) compared to illiterate ones (2.8%). Additionally, the infection rate was lower among employed individuals (5.4%) compared to unemployed participants (11.9%).

The burden of GC infection in males (9.5%) was higher than in females (7.9%). The odds of GC infections in males were found to be 4.12 times higher than in females (AOR = 4.12, 95% CI: (0.26–0.65). The prevalence of GC infections in rural residents (9.3%) was higher than in urban residents (8.1%). Those participants who were rural dwellers had 2.34 times higher GC infection than their counterparts (AOR = 2.34, 95% CI: 48–3.69) (Table 1).

### Factors associated with *N. gonorrhoeae* infections

Of the respondents, 163 (28.5%) were used condoms, 151(26.4%) bought condom, and 121 (22.2%) were had a condom in their home. One hundred sixty-eight (29.4%) were previous STI drug users, 211(37.0%) had a previous STI, and (193) (33.8) had STI in the last five years (Table 2).

**Table 2:** Factors associated with *N. gonorrhoeae* infections among STI-suspected patients (*n* = 571) in Addis Ababa, Ethiopia, 2024.

Socio-demographic	*N. gonorrhoeae* infection		COR (95% CI)	*P*-value	AOR (95% CI)	*P*-value
Characteristics *n* (%)	Positive (*n* = 99) *n* (%)	Negative *n* = 472 *n* (%)				
Living with family						
Yes (188) (32.9)	32 (5.6)	156 (27.3)	0.97 (0.61–1.54)	0.889		
No (383) (67.1)	67 (11.7)	316 (55.3)	1.00			
Condom use						
Yes (163) (28.5)	32 (5.6)	131 (22.9)	1.00			
No (408) (71.5)	67 (11.7)	341 (59.7)	1.24 (0.78–1.98)	0.361		
Buy condom						
Yes (151) (26.4)	32 (5.6)	119 (20.8)	0.71 (0.44–1.13)	0.146	0.96 (0.557–1.65)	0.884
No (420) (73.6)	67 (11.7)	353 (61.8)	1.00			
Condom at home						
Yes (121) (21.2)	24 (7.5)	97 (17.0)	1.24 (0.74–2.01)	0.414		
No (450) (78.8)	99 (17.3)	375 (65.7)	1.00			
Previous STI Drug use						
Yes (168) (29.4)	41 (7.2)	127 (22.2)	1.92 (1.23–3.01)	0.004	1.41 (1.18–1.91)	0.029
No (403) (70.6)	58 (10.2)	345 (60.4)	1.00			
Previous STI						
Yes (211) (37.0)	45 (7.9)	166 (29.1)	1.54 (0.99–2.38)	0.055	1.38 (0.56–3.40)	0.489
No (360) (63.0)	54 (9.5)	306 (53.6)	1.00			
Using injectable drugs						
Yes (154) (27.0)	28 (4.9)	126 (22.1)	1.08 (0.67–1.76)	0.75		
No (417) (73.0)	71 (12.4)	346 (60.6)	1			
Sexual Partner >1						
Yes (243) (42.6)	53 (9.3)	190 (33.3)	1.71 (1.11–2.64)	0.016	1.16 (0.62–2.20)	0.634
No (328) (57.4)	46 (8.1)	282 (49.4)	1			
Early sexual Intercourse						
Yes (46) (8.1)	9 (1.6)	37 (6.5)	1.18 (0.55–2.52)	0.678		
No (525) (91.9)	90 (15.8)	435 (76.2)	1			
New sexual Partner						
Yes (257) (45.0)	60 (10.5)	197 (34.5)	2.15 (1.38–3.34)	0.001	0.74 (0.36–1.52)	0.408
No (314) (55.0)	39 (6.8)	275 (48.2)	1			
Sex with commercial sex worker						
Yes (233) (40.8)	55 (9.6)	178 (31.2)	2.06 (1.33–3.12)	0.02	1.14 (0.59–2.19)	0.342
No (338) (59.2)	44 (7.7)	294 (51.5)	1			
Watch pornographic film						
Yes (207 )(36.3)	42 (7.4)	165 (28.9)	1.37 (0.88–2.13)	0.016	1.8 (1.01–3.28)	0.046
No (364) (63.7)	57 (10.0)	307 (53.8)	1			
Attending night club						
Yes (274) (48.0)	62 (10.9)	212 (37.1)	2.05 (1.32–3.21)	0.001	1.26 (0.70–2.27)	0.43
No (297) (52.0)	37 (6.5)	260 (45.5)	1			
Alcohol use						
Yes (373) (65.3)	82 (14.4)	291 (51.0)	3.0 (1.72–5.22)	0.001	1.35 (1.196–1.62)	0.001
No (198) (34.7)	17 (3)	181 (31.7)	1			
Khat use						
Yes (249) (43.6)	59 (10.3)	190 (33.3)	2.20 (1.41–3.41)	0.001	1.55 (1.32–1.95)	0.001
No (322) (56.4)	40 (7.0)	282 (49.4)	1			
Smoking cigarettes						
Yes (176) (30.8)	31 (5.4)	145 (25.4)	1.01 (0.64–1.64)	0.908		
No (395) (69.2)	68 (11.9)	327 (57.3)	1			
Religious education						
No (319) (55.9)	67 (11.7)	252 (44.1)	1.83 (1.16–2.89)	0.001	1.70 (1.03–2.88)	0.04
Yes (252) (44.1)	32 (5.6)	220 (38.5)	1			
Prolonged chronic disease						
Yes (49) (8.6)	13 (2.3)	36 (6.3)	1.34 (0.93–3.58)	0.079	1.55 (0.26–2.167)	0.521
No (522) (91.4)	86 (15.1)	436 (76.4)	1			

Bivariate and multivariable logistic regression were used to identify the possible risk factors for the infection of *N. gonorrhoeae*. The odds of *N. gonorrhoeae* infection were found to be 1.41 times higher among the previous drug users than the nonusers [AOR = 1.41, 95% CI: 1.41(1.18–1.91)]. Those participants who were watching pornographic films were more vulnerable to *N. gonorrhoeae* infections than their counterparts (AOR = 1.8, 95% CI: (1.01–3.28); however, those who had more sexual partners, new sexual partners, had sex with a commercial sex worker, and had an early sexual intercourse history were not statistically associated with *N. gonorrhoeae* infections. Participants who had an alcohol use history were statistically associated with *N. gonorrhoeae* infections [AOR = 1.35, 95% CI: 1.35 (1.196–1.62)]. Moreover, the odds of *N. gonorrhoeae* infection were 1.55 times higher among chat users than the non-chat users [AOR = 1.55, 95% CI: (1.32–1.95)], however, having a smoking cigarette history was not statistically significant with *N. gonorrhoeae* infection. Participants who didn't attend the religious sectors were more vulnerable to the infection than their counterparts [AOR = 1.70, 95% CI: (1.03–2.88)] (Table 2).

### Prevalence of risky sexual behavior

Of the male respondents, 157 (27.5%) were having sexual intercourse. Being males were statistically more significant than females for RSB (*p* < 0.001). Of the participants, the likelihood of being sexually active rose with the increase of age. At the age of 15–29 (0.4%), 25–29 (16.3%), 30–34 (13.5%), and >35 (17.7%) reported having sex with two or more however, age were not statistically significant with RSB. One hundred ninety-five (34.2%) respondents among urban had two or more sexual partners and 250(43.8%) of literates had a sexual history with two or more (*p* < 0.001). Moreover, 222(38.9%) of the employers had two or more sexual partners (*p* < 0.001). The prevalence of RSB among STI patients was 56.9% (Table 3).

**Table 3:** Socio-demographic characteristics of the study participants with risky sexual behavior among STI-suspected patients (*n* = 571) in Addis Ababa, Ethiopia, 2024.

Socio-demographic	Risky sexual behavior (RSB)		COR (95% CI)	*P*-value	AOR (95% CI)	*P*-value
Characteristics *n*	Positive (*n* = 325) *n* (%)	Negative (*n* = 246) *n* (%)				
Gender						
Male (216)	157 (27.5)	59 (10.3)	2.96 (2.05–4.26)	0.001	0.35 (0.23–0.53)	0.002
Female (355)	168 (29.4)	187 (32.7)	1			
Age						
18–19 (8)	2 (0.4)	6 (1.1)	3.69 (0.72–18.79)	0.115		
20–24 (71)	52 (9.1)	19 (3.3)	0.45 (0.25–0.82)	0.009		
25–29 (139)	93 (16.3)	46 (81.)	0.61 (0.38–0.96)	0.034		
30–34 (170)	77 (13.5)	93 (16.3)	1.48 (0.97–2.26)	0.064		
>35 (183)	101 (17.7)	82 (14.4)	1			
Resident						
Urban (352)	195 (34.2)	157 (27.5)	0.85 (0.60–1.19)	0.353		
Rural (219)	130 (22.8)	89 (15.6)	1			
Marital status						
Unmarried (320)	241 (42.2)	79 (13.8)	0.16 (0.11–0.24)	0.001	0.16 (0.11–0.24)	0.001
Married (251)	84 (14.7)	167 (29.2)	1			
Educational status						
Illiterate (112)	75 (13.1)	37 (6.5)	1.69 (1.09–2.62)	0.017	0.45 (0.27–0.75)	0.001
Literate (459)	250 (43.8)	209 (36.6)	1			
Occupation						
Unemployed (329)	222 (38.9)	107 (18.7)	2.80 (1.98–3.94)	0.001	0.41 (0.27–0.59)	0.001
Employed (242)	103 (18.0)	139 (24.3)	1			

**Figure 2:**
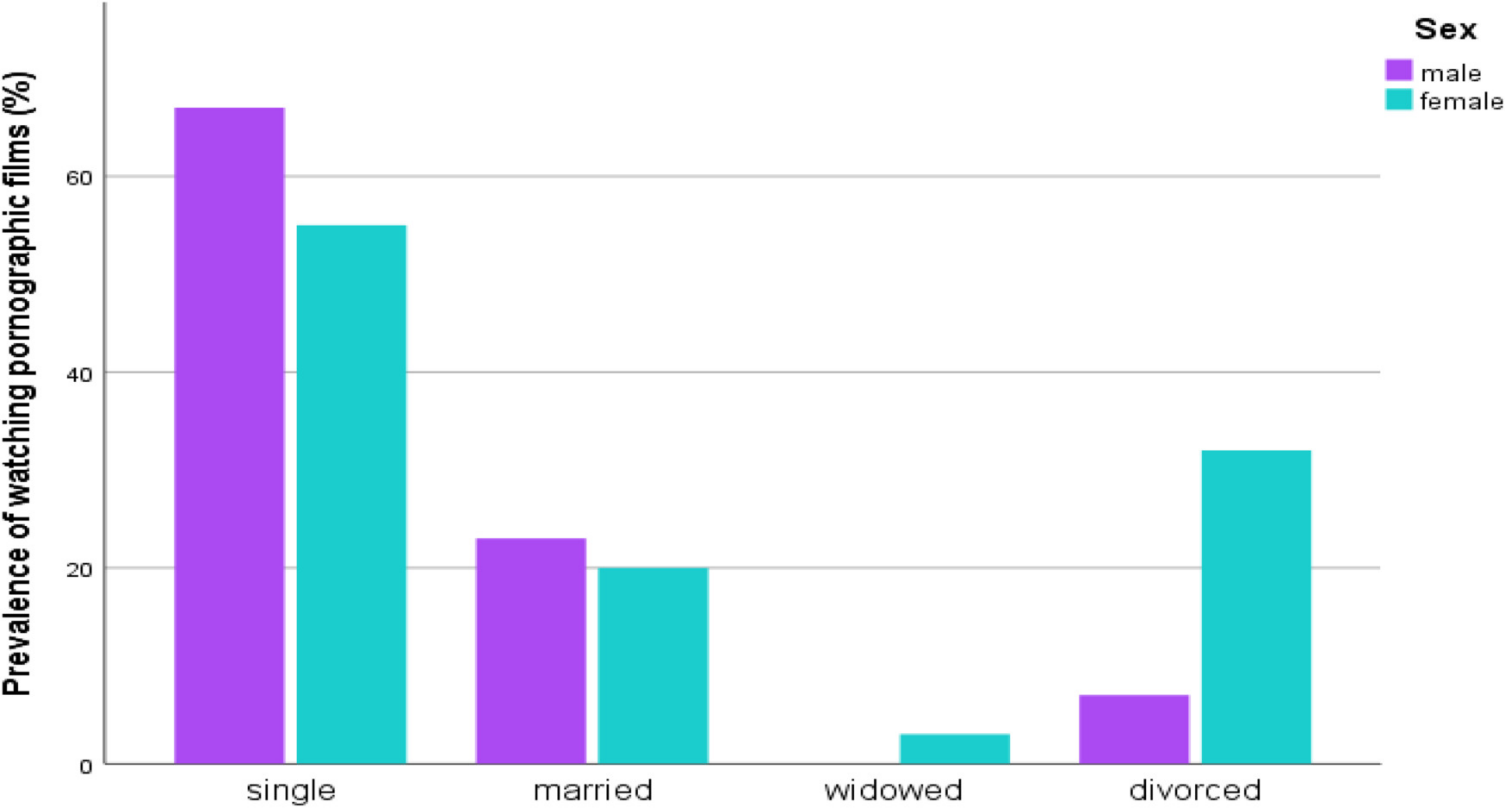
Distribution of watching pornographic films by marital status and sex of STI suspected patients in Addis Ababa, Ethiopia.

The percentage of participants watching pornographic films among study subjects was 36.3% (*n* = 207). Of these participants, females (53.1%, *n* = 110) had more experience watching the films than males (46.9%, *n* = 97). Among the 207 study subjects who had experience watching pornographic films, a higher level of experience was observed in singles (58.9%, *n* = 122), followed by married individuals (20.8%, *n* = 43), and divorced individuals (18.8%, *n* = 39), compared to the widowed group (1.4%, *n* = 3) (Figure 2).

### Factors influencing the acquisition of risky sexual behavior

One hundred eighty-eight respondents were living with their family; of them, 89 (15.6%) had an RSB. Of the positive RSB response individuals, 127 (22.2%) used condoms during sexual intercourse, 118 (20.7%) bought condoms, and 94 (16.5%) had a condom at their home. Moreover, 145 (25.4%) had a previous history of usage of STI drugs, 182 (31.9%) had a previous history of STI (*P* < 0.001), and 164 (28.7%) had a history of STI within the last five years.

The odds of having multiple sexual partners and early sexual intercourse were 10.95 (95% CI 5.75–20.84) times higher among STI-suspected patients who had a new sexual partner than their counterparts. The odds of risky sexual behavior were 3.31 times higher in those attending nightclubs than their counterparts [AOR = 3.31, 95% CI (1.82–1.90)] and also religious education had a positive impact on the prevention of risky sexual behavior [AOR = 0.5, 95% CI (0.29–0.86)]; however using a chat 59 (10.3%), smoking cigarette 144 (25.2%) and having a prolonged chronic disease 36 (6.3) hadn't had a statistically significant association with risky sexual behavior.

Study participants who had a previous STI history were 2.85 times [AOR = 1.58, 95% CI (1.17–6.91)] more likely to have a risky sexual behavior than those who didn't have a previous STI disease history. Similarly, study subjects who watched pornographic films regularly were also more exposed to risky sexual behavior than those who weren't involved in watching pornographic films [AOR = 2.32, 95% CI (1.84–5.14)] (Table 4).

**Table 4:** Factors associated with risky sexual behavior among STI-suspected patients (*n* = 571) in Addis Ababa, Ethiopia, 2024.

Socio-demographic characteristics	Risky sexual behavior		COR (95% CI)	*P*-value	AOR (95% CI)	*P*-value
	Positive (*n* = 325) *n* (%)	Negative (*n* = 246) *n* (%)				
Living with family						
No (383)	236 (41.3)	147 (25.7)	0.56( (0.39–7.97)	0.046	0.85 (0.61–1.18)	0.322
Yes (188)	89 (15.6)	99 (17.3)	1			
Condom use						
No (408)	198 (34.7)	210 (36.8)	0.26 (0.17–0.41)	0.001	1.16 (0.49–2.71)	0.731
Yes (163)	127 (22.2)	36 (6.3)	1			
Buy condom						
No (420)	207 (36.3)	213 (37.3)	0.27 (0.17–0.42)	0.001	0.41 (0.15–1.16)	0.093
Yes (151)	118 (20.7)	33 (5.8)	1			
Condom at home						
No (450)	231 (40.5)	219 (38.4)	0.30 (0.19–0.48)	0.034	2.26 (.77–6.62)	0.137
Yes (121)	94 (16.5)	27 (4.7)	1			
Previous STI drug use						
Yes (168)	145 (25.4)	23 (4.0)	7.81 (4.82–12.64)	0.016	1.40 (0.52–3.77)	0.505
No (403)	180 (31.5)	223 (39.1)	1			
Previous STI						
Yes (211)	182 (31.9)	29 (5.1)	9.52 (6.102–14.86)	0.001	2.85 (1.17–6.91)	0.021
No (360)	143 (25.0)	217 (38.0)	1			
New sexual partner						
Yes (257)	60 (10.5)	197 (34.5)	2.148 (1.37–3.34)	0.001	10.95 (5.75–20.84)	0.001
No (314)	39 (6.8)	275 (48.2)	1			
Watch pornographic films						
Yes (207)	181 (31.7)	26 (4.6)	0.09 (0.059–0.149)	0.001	2.32 (1.84–5.14)	0.001
No (364)	144 (25.2)	220 (38.5)	1			
Attending night club						
Yes (274)	62 (10.9)	212 (37.1)	2.055 (1.32–3.21)	0.001	3.31 (1.82–1.90)	0.001
No (297)	37 (6.5)	260 (45.5)	1			
Alcohol use						
Yes (373)	82 (14.4)	291 (51.0)	3 (1.72–5.22)	0.001	3.32 (1.90–5.78)	0.001
No (198)	17 (3.0)	181 (31.7)	1			
Khat use						
Yes (249)	59 (10.3)	190 (33.3)	2.189 (1.41–3.41)	0.021	1.24 (0.60–2.55)	0.558
No (322)	40 (7.0)	282 (49.4)	1			
Cigarette						
Yes (176)	144 (25.2)	32 (5.6)	5.32 (3.46–8.19)	0.031	0.85 (0.38–1.88)	0.322
No (395)	181 (31.7)	214 (37.5)	1			
Religious education						
Yes (252)	32 (5.6)	220 (38.5)	1.83 (1.15–2.89)	0.001	0.50 (0.29–0.86)	0.012
No (319)	67 (11.7)	252 (44.1)	1			
Prolonged chronic disease						
Yes (49)	13 (2.3)	36 (6.3)	1.72 (0.93–3.39)	0.079	2.36 (0.74–7.53)	0.146
No (522)	86 (15.1)	436 (76.4)	1			

## Discussion

STIs are one of the major public health problems. Reducing the STI burden is one of the WHO's third Sustainable Development Goals, and it is planned to minimize NG infection by 90% globally by 2030 (30). The WHO plans to develop new methods for the diagnosis, prevention, management, and epidemiology of STIs, including NG. They plan to design a new detection test that have a low-cost, rapid, and accessible test with self-sampling and detection to reduce the transmission rate, incidence, and prevalence of NG infection, which is especially helpful in the low-resource settings of developing countries where the burden of the disease is high (31).

The present study aimed to determine the distribution of NG infections among suspected STI patients, which helps to reduce the impact of disease causations and improve public health.

In this study, the prevalence of *N. gonorrhoeae* among suspected STI patients was 17.3%, which was higher than studies done in Mekele (10.04%) (9), Jimma (9.8%) (13), Gondar (7.6%) (10), Hawassa (3.3%) (32), Kenya (6.3%) (33), India (5.7%) (34), Latin America in low-risk groups (1.46%), and high-risk groups (5.68%) (35), and WHO European region (1.0%) (6). This relatively increased rate of gonococcal infections seen in the present study might be due to multicultural practice in the city where commercial sex work was high (22, 36). The city is the capital city, it is the center for business, education, employment, medication, and it is also a transition center to travel from one area to another, and residence of many people coming from different corners of the country to the city including migrant workers displaced from rural and urban areas of other regions of Ethiopia (37, 38). Additionally, areas with rare male circumcision practices are also represented in the city, with a prevalence of 53% of males not circumcised in the Gambela region (39). However, the association between male uncircumcision and the development of NG infection has not been adequately studied. Studies have shown that uncircumcised men are more likely to develop NG infection than circumcised men (40, 41). Such practices increase the risk factors for acquiring STI and *N. gonorrhoeae* infections. The relative increase of gonococcal infections in the present study area may also indicate the presence of HIV (23, 42) and other STIs that increase the risk of acquiring *N. gonorrhoeae*. In contrast, the prevalence in the present study was lower than in studies done in Addis Ababa (69%) (8), Ethiopia (20%) (7), Ghana (27.4%) (43), and Nigeria (19.1%) (44). The difference could be variations in study populations, geographical differences, sample size, specimen type, and social beliefs.

In the present study, the overall magnitude of risky sexual behavior among sexually transmitted infections was 56.9%, which was lower than reports found in Haramaya University students (65.8%) (45), Southern Ethiopia (75.5%) (46) and Southern Ethiopia (78%) (47). On the other hand, the finding was much higher than studies done at Injibara University (38%) (48), Ethiopian public university students (19.5%) (49), Southern Ethiopia (25.9%) (50), Gondar (27.5%) (51) and Addis Ababa (40.6%) (52). The variations could be differences in the target study populations.

According to the present findings, culture-confirmed positive *N. gonorrhoea*e isolates were higher in males than in females, and being male showed a statistically significant association for *N. gonorrhoeae* infections and risky sexual behavior. A study done in Uganda (17) also supports these findings. This could be because females might have been more likely to have public clinic follow-ups that enabled them to know STI prevention and control methods.

The present study showed that RSB was higher among the non-employed than the employed. The study was supported by the previous studies done in Addis Ababa (52). This might be due to an individual who hadn't employed maybe feeling more levels of independence and less levels of social expectations and responsibilities, which can lead to having multiple sexual partners and committing sexual intercourse. The educational levels of the participants were also statistically significantly associated with the RSB. Individuals who were not educated were more at risk for RSB than those who were educated, which was supported by studies done in Gondar (51), Addis Ababa (52), and Southern Ethiopia (47). That might be due to illiterates having less awareness of sexually transmitted diseases.

In the present study, marital status was not statistically associated with the prevalence of *N. gonorrhoeae* infection; however, being unmarried was statistically significantly associated with risky sexual behavior. In contrast, a study done in Jimma (13) on sexually transmitted disease patients indicated that marital status was significantly associated with the prevalence of *N. gonorrhoeae* infections. Additionally, the current study showed that respondents who were rural residents were more likely to have *N. gonorrhoeae* infections than urban dwellers, which was supported by a study done at Jimma (13), whereas being urban and rural were not statistically different for risky sexual behavior. This difference might be due to geographical location variations; being urban has a chance of getting information from health professionals regarding the negative effects of risky sexual behavior and the burden of sexually transmitted infections.

The current study indicated that respondents who had a previous STI drug use history had significant associated risk factors for the acquisition of *N. gonorrhoeae* infection than those who hadn't had a history of STI drug use, but having a previous drug use history was not a risk factor for the RSB. However, individuals who had a previous STI and who didn't take the drug were statistically significant with RSB, which was supported by a study done in Mekele (9).

In our findings, the odds of risky sexual behavior and *N. gonorrhoeae* infection were higher among STIs who drank alcohol as compared with STIs who didn't drink alcohol. Similar findings were reported in the previous studies of Gondar (10) and India (34). Also, drinking alcohol was a statistically significant factor for the occurrence of risky sexual behavior, which was supported by studies done in Addis Ababa (52) and Southern Ethiopia (50). That could be due to excessive drinking of alcohol, which affects personal judgment and behavior, altering decisions, and creating a conflict of interest between desire and inhibitions that leads to risky sexual practices.

The use of khat chewing was another risk factor in the present study. The finding indicated that individuals who chewed khat were at 1.55 times higher risk of engaging in N*. gonorrhoeae* infections than non-khat chewers, which has been supported by studies done in Mekele (9). However, chewing khat was not an associated factor for risky sexual behavior in contrast with this a study done in Bahir dar city showed that chewing khat was a factor for risky sexual behavior (53).

According to our report, there was a positive association between religious beliefs and practices and a lower prevalence of *N. gonorrhoeae* infections and RSB. This finding was supported and stated that adolescents and young adults who associated with their religion throughout their lives had shown lower rates of RSB, which was supported by studies done in (54, 55) and *N. gonorrhoeae* infections. Religious practice had a protective effect on risky sexual behavior.

The current study showed that study participants who watched pornographic movies were more likely to have *N. gonorrhoeae* infections and RSB as compared with their counterparts, which was supported by a study done in Addis Ababa (52) and Southern Ethiopia (47). Similarly, attending nightclubs was also a statistically significant association with risky sexual behavior, but not for *N. gonorrhoea*e compared with those who weren't. Studies done in Gondar (51) and Southern Ethiopia support this finding (50). It could be due to watching pornographic films, and attending nightclubs may increase the motivations for sexual needs that were prone to risky sexual behavior.

## Conclusion

The prevalence of *N. gonorrhoeae* and risky sexual behavior among STI patients in Addis Ababa was high. Gender, watching pornographic films, alcohol consumption, and not participating in religious education have been found to increase the risk of experiencing both *N. gonorrhoeae* infections and risky sexual behavior. Moreover, a previous drug use history, residence, and chat chewing were the risk factors for the prevalence of *N. gonorrhoeae* infections, whereas marital status, educational status, occupational status, having a previous STI, and having a new sexual partner were the risk factors for risky sexual behavior. Therefore, health education has to be given to the community to create awareness of sexuality and risky sexual behaviors, as the risk factors indicate that knowledge and attitude gaps exist.

## Limitations of the study

The limitations of the present study were important factors like social norms, other risky sexual behaviors, and beliefs that were not included, which would have an impact on risky sexual behavior and sexually transmitted diseases. Moreover, because of the sensitive nature of risky sexual behavior and sexually transmitted diseases, response bias of the participants may be present, and the data in the present study were not also nationally representative. Therefore, further studies should be undertaken at the national level and include important factors to explore more on *N. gonorrhoeae* and risky sexual behavior.

## Data Availability

The original contributions presented in the study are included in the article/Supplementary Material, further inquiries can be directed to the corresponding author.
